# Exploring Youth Perceptions About Cancer Prevention and Preferences for Education: a Qualitative Study

**DOI:** 10.1007/s13187-021-02077-0

**Published:** 2021-08-13

**Authors:** Olufunmilola Abraham, Lisa Szela, Emilie Feng, Maryann Egbujor, Sommer Gay

**Affiliations:** 1 Social and Administrative Sciences Division , University of Wisconsin-Madison School of Pharmacy, 777 Highland Avenue, Suite 2515, Madison, USA; 2 Social and Administrative Sciences Division, University of Wisconsin-Madison School of Pharmacy, Madison, WI 53705 USA

**Keywords:** Cancer knowledge, Adolescent knowledge, Adolescent education, Cancer education, Cancer awareness, Behavior, Attitudes

## Abstract

This study aimed to characterize adolescents’ perspectives on cancer and cancer prevention and to explore their preferences for cancer education. A total of 188 middle and high school students participated in 25 focus groups. Focus groups were led by study team members and included five to ten participants each. Adolescents were asked to discuss their cancer and cancer prevention knowledge, sources of cancer education, and preferences for cancer prevention education. Focus groups were audio-recorded, and professionally transcribed. Transcripts were content and thematically analyzed by two study team members using NVivo qualitative data software. Six themes were identified in focus groups: knowledge about cancer, negative perceptions of cancer, awareness of cancer prevention, engagement in cancer prevention, facilitators and barriers for action, and preferences for cancer education. Adolescents described a wide range of cancer education sources, including friends, family, healthcare professionals, school, technology, and pamphlets. Participants associated cancer with negative emotions, such as sadness and fear. Participants expressed interest in learning about cancer and cancer prevention, but also identified barriers to engaging in preventative behaviors, such as discomfort, lack of knowledge, and lack of personal connection to cancer. Adolescents preferred learning through personal presentations, online videos, school, educational games, and social situations. Adolescence is a critical period for learning and developing healthy behaviors. Awareness of cancer and cancer prevention is crucial to lowering cancer risk in the future. To be effective, cancer education for adolescents should target specific areas of cancer prevention and address barriers to engagement in preventative behaviors.

**Supplementary Information:**

The online version contains supplementary material available at 10.1007/s13187-021-02077-0.

## Introduction

Cancer is the second leading cause of death worldwide [1]. The World Health Organization (WHO) estimates that cancer deaths are likely to surpass that of ischemic heart disease, the leading cause of death worldwide, in the next four decades [1]. Additionally, cancer poses a dramatic clinical burden, disrupts social standards, and erodes many economic resources [1]. People of all ages, genders, and race can be affected by this disease, including adolescents [2]. The Centers for Disease Control and Prevention (CDC) estimates that the overall cancer incidence rate among adolescents and young adults rose by 0.9% on average per year during 2012 to 2016 [3].

Awareness is key to identifying personal risk factors and preventing cancer at an early age. However, studies demonstrate that adolescents are not well informed about cancer risk factors [4]. Current literature suggests that many adolescents and college students in the USA lack cancer-preventative knowledge and engage in cancer risk behaviors such as unhealthy diet, frequent alcohol consumption, and low physical activity [5, 6]. In one study, only 49% of adolescents reported awareness of the human papillomavirus (HPV) vaccine and cervical cancer [7]. Addressing this lack of awareness is crucial to empowering adolescents to make choices to prevent cancer and reduce cancer risk factors.

Awareness of modifiable risk factors is an important starting point in the promotion of positive health outcomes [4]. A systematic review showed six trials involving educational interventions for adolescents reported a positive effect on composite sun protection behaviors [8]. Another study using a Cancer Awareness Measure (CAM) showed improvement of cancer warning signs within two weeks of the intervention and a statistically significant decrease in the percentage of adolescents who reported that they did not know cancer risk factors [9]. Moreover, studies show early interventions can reduce the long-term impact of unhealthy behaviors acquired in adolescence [10]. Educating adolescents about cancer prevention is a crucial step to improving the number of cancer-related deaths each year.

Adolescence provides a window of opportunity for cancer risk education and intervention. Cancer prevention and education should be targeted at this group because they are in an age of active learning but are also in a stage where risky behaviors, such as smoking, begin [11]. Data suggests that adolescents are under-informed about the relationship between health behaviors and cancer risk [12]. Although improving cancer awareness among adolescents is critical for life-long patterns of healthy behavior, little is known about adolescents’ perceptions of cancer risk factors and their preferences for receiving cancer education [4]. The aim of this study was to characterize adolescents’ perceptions about cancer and cancer prevention and to understand their preferences for receiving cancer prevention education.

## Methods

### Study Design

The study team developed a focus group guide consisting of questions that explored adolescents’ perceptions and knowledge of cancer as well as cancer prevention and preferences for cancer education (see appendix). The guide consisted of open-ended questions divided into sections pertaining to cancer knowledge, cancer prevention knowledge, cancer education, and cancer prevention education. Most focus group questions were generated by the study team or adapted from *Cancer- Educate to Prevent* [13]. Two questions about participants’ perceptions of cancer were modified from a study regarding mothers’ and high school students’ perceptions of cervical cancer, human papillomavirus (HPV), and the HPV vaccine [14]. The study team used feedback from the University’s Survey Center to revise the guide for content and clarity. Focus groups were chosen to capture group interaction and discussion and to allow participants to expand on their responses and opinions. This qualitative methodology provided a collaborative environment for participants to build on each other’s responses and for focus group facilitators to ask follow-up questions as needed.

### Recruitment

Adolescents were recruited from one middle school and one high school in Wisconsin from January to February 2020. Students were eligible if they were enrolled in grades 7 to 12 and could speak and understand English. School staff distributed packets containing a letter of introduction to the study, consent forms, and a request to return completed forms on a later date. All consent documents were available in English and Spanish for adolescents with Spanish-speaking parents or guardians. One school distributed the recruitment packets to all students in a required health class, and one school made recruitment packets available to a specific science class. Parental consent and student assent were required for participants under the age of 18; students aged 18 and older were able to consent and participate without parental consent. Participants were each given $10 in cash as an incentive for participation. This study was approved by the University’s Institutional Review Board.

### Data Collection

Each focus group consisted of five to ten participants, one facilitator from the study team, and one to two other study team members as moderators. One study team member led the focus group discussion, while the other moderator(s) took observation notes and asked clarifying questions as needed. To ensure privacy and confidentiality for participants, schools provided separate rooms for each focus group, and participants were encouraged not to share identifying information. Each focus group lasted approximately 35 to 50 min, was audio-recorded, and professionally transcribed verbatim. All identifying information was redacted from the transcripts before analysis. Participant demographic information was collected via paper survey at the end of each focus group. Focus group facilitators and moderators debriefed and created reflection notes following each session.

### Data Analysis

Two members of the study team independently verified focus group transcripts for accuracy before beginning data analysis. Transcripts were content and thematically analyzed by two study team members using NVivo 12 (QSR) qualitative software. Each study team member reviewed all transcripts to develop relevant codes using both an inductive and deductive approach. Relevant codes were then combined to create the master codebook. The study team held biweekly meetings to discuss codes, review the master codebook, and coding structure, and address discrepancies. Microsoft Excel was used to determine the intercoder reliability, and the final average Kappa score was 0.86. Thematic analysis of prevalent codes was completed using Microsoft Excel, and codes were categorized into major themes and subthemes.

## Results

Study participant characteristics are described in Table 1. Verbatim quotes are provided in Table 2.

**Table 1:** Participant demographics

**Demographics**	*N*	%
**Age (y)**		
12–13	89	47.3
14–15	68	36.2
16–18	31	16.5
**Gender**		
Male	85	45.2
Female	103	54.8
**Level of education**		
Middle school	157	83.5
High school	31	16.5
**Race/ethnicity**		
American Indian or Native American	1	0.5
Asian	6	3.2
Black or African American	4	2.1
Hispanic or Latino	7	3.7
Other^a^	1	0.5
Reported more than one race/ethnicity	14	14
White	155	82.4
**Number of youths at home (excluding the participant)**		
1–2	110	58.5
3–4	64	34
5 or more	14	7.4

^a^Participants were given the option to select “Other” for race/ethnicity

**Table 2:** Themes, subthemes, and verbatim quotes

Theme	Subtheme	Verbatim quotes
Knowledge about cancer	Sources of cancer knowledge	“There’s also like a lot of cancer pamphlets, like, you know, at the doctor’s office, like schools sometimes, like libraries, a bunch of other resource places, where you can read about it. And that’s like how I kind of know about some stuff.” – FG 2
		“I’ve learned it from like sometimes in schools, but it’s never really like a huge thing. But I’ve learned it mostly from like the media in general, like news and like other things, like commercials and like campaigns.” – FG 15
	Unstructured cancer learning experiences	“You just like learn about it everywhere, like family, friends, like teachers, pretty much like everywhere you go, like a little bit of information about it.” – FG 3
		“I kind of just learn about it from hearing what goes on, kind of. You just hear it out in the open that you kind of just pick up on it.” -FG 10
	Knowledge about the causes of cancer	“Yeah. I think like my grandma and my dad both had large B-cell lymphoma, and it’s like genetically in my family. So like I know a lot about that and like risks for that too.” – FG 30
		“And it’s also heavily like influenced by, like, yes, your genetics but also the way that your environment is and the way your lifestyle, like how you live. And it’s just like there’s a ton of different factors that go into it that, which is what makes it hard to really identify, truly, what it is.” – FG31
	Awareness of cancer types and cancer statistics	“Leukemia, lung cancer, skin cancer, breast cancer, those are the ones that I know a lot about.” – FG 13
		“I’ve heard of like breast cancer, bone cancer. And like I know breast cancer can, a lot of times, spread to other parts of your body, and then bone cancer is pretty fatal because like it breaks down the, your bones.” – FG 14
	Knowledge of cancer diagnosis and treatment	“I think of chemotherapy because it’s like stuck in me that they put radioactive material through you.” – FG 16
		“Usually, it’s just like you see a mole. You can cut it out. Like you’re usually okay. And then there’s like pancreatic, which is really hard to catch, so normally, it’s really late stage. Well, pancreatic isn’t even measured in stages. And then like brain cancer is pretty fatal.” – FG 31
Negative perceptions of cancer		“I think of a really emaciated person lying in a hospital bed about to die.” – FG 13
		“I think of [cancer as] scary or can kill you, in a way, and ruin your life.” – FG 21
Awareness of cancer prevention		“Drinking and smoking and like chewing tobacco. Stuff that usually harms your body can be, like smoking can cause lung cancer or something like that.” – FG 7
		“I guess like knowing your family history and if you’re more like at risk than other people would help too because then you’d know what to be looking out for and what to be trying to avoid.” – FG 34
Engagement in cancer prevention	Interest in learning about cancer and cancer prevention	“I feel like some people would care, but some wouldn’t. And it might just turn out being like another lesson in school that you learn about and then like take a test on but then not really sure about it. Like it’s important, but that just might be how it turns.” – FG 1
		“I think it just depends because I feel like if someone in their family has had cancer, then they’d be more interested in learning about it. Then if maybe you’ve never really experienced what cancer can do, then maybe you wouldn’t be so interested.” – FG 14
	Engagement in cancer prevention behavior	“Well, I just like don’t even do any of the smoking, drugs, alcohol stuff just because I know it’s bad for you. And then the, like being in the sun, I try to put on sunscreen whenever I go outside for a good, long period of time.” – FG 4
		“I guess I always think about skin cancer when I put on sunscreen, which is a little weird, but because my dad had melanoma, and they like tracked it back to like he got this like really bad sunburn once.” – FG 30
	Attitudes toward cancer prevention	“Well, I’m pretty sure no one wants cancer, so it’s important when you think about like all the things that can help prevent it and things that can help cause it and how you can avoid it.” – FG 2
		“I think it’s very important to make sure that you can do everything you can because nothing is 100%. But you really have to try and make sure that you can do what you can so that you don’t get sick.” – FG 8
Facilitators and barriers for action	Facilitators—reasons for learning about cancer or engaging in preventative behavior	“Well, I think people just like, we’ve seen the effects before. And like especially people who have had younger siblings or like people who like it’s your job to take care of, like we want to make sure that we’re well informed so that not only like we make sure that we don’t get sick but like they don’t get sick, you know. Like we can lower anyone’s chances who we’re close to.” – FG 2
		“I definitely try to avoid smoking and drinking as well, kind of just like not just for cancer but like just being healthy in general.” – FG 34
	Barriers—limiting factors to cancer education or preventative behavior	“Yeah. It’s like if you’re not affected by it, it would be kind of like, well, it doesn’t affect me, so it’s not really a big deal in my eyes.” – FG 10
		“But it’s, it always slips your mind and too, and you notice it with other people that, you know, you just forget to put on sunscreen, and then you’re out in the sun for hours on end, and you have no protection.” – FG 30
Preferences for cancer education	Method of learning about cancer	“It depends on who’s like giving the video because there’s a lot of influential people out there that people would rather watch videos on than like these weird doctors that they probably wouldn’t really trust or find interesting.” – FG 17
		“I think for me, learning about cancer in a classroom setting makes it feel less real to me, whereas if I hear it from family or friends, it feels like it’s a more pertinent issue.” – FG 34
	Learning online and misinformation	“If I knew it was official, like this is the official account of Hospital A, I would trust it and maybe look into it a bit. But it’s just like I have no idea, and it would be kind of edgy.” – FG 12
		“They could be telling something that’s not true. So I wouldn’t really trust it if it’s... not Mayo Clinic or something like that.”- FG 13
	Desired features of cancer education	“As long as the video isn’t like super long because if I go onto a video and I notice that it’s like really long, I’m just like, I’m not going to listen to this whole thing.” – FG 7
		“I feel like if you focus it more to like our age group and make it a little more interesting to what we want to hear, and like it still can be about cancer but like in a more fun activity way to help us learn about it. Then kids might want to learn about it more.” – FG 14

A total of 188 adolescents participated in 25 focus groups. Participants ranged in age from 12 to 18 years old and grades 8 to 12. Participants were 54.8% female, 89.4% white, and 83.5% middle school students. Six major themes were identified including (1) knowledge about cancer, (2) negative perceptions of cancer, (3) awareness of cancer prevention, (4) engagement in cancer prevention, (5) facilitators and barriers for action, and (6) preferences for cancer education.

### Knowledge About Cancer

#### Sources of Cancer Knowledge

Participants reported being exposed to cancer content and topics from a wide range of sources. Sources included parents, family members, friends, doctors, and people who had been diagnosed with cancer. Exposure to cancer content also occurred at schools, libraries, and hospitals. A variety of information sources were cited, such as television, books, the news, pamphlets or posters, podcasts, social media, websites, and online videos.

#### Unstructured Cancer Learning Experiences

Some participants could not define a specific source for their cancer knowledge and described “hearing about cancer” from others. Participants described the process as confusing, stating that they learned “bits and pieces” rather than receiving in-depth information.

#### Knowledge About the Causes of Cancer

Participants described cancer as physiological, behavioral, and multifactorial in origin. They identified physiological factors, such as changes in cell development, metastasizes, and the role of genetics in cancer development. Some participants defined cancer as an illness, disease, and cause of mortality, “a disease that happens when like, cells start growing uncontrollably”, or “mutation of the cells.” Cancer was described as multifactorial, with multiple causes and risk factors. Participants stated that actions such as engaging in preventative behavior and participating in screenings could lower cancer risk.

#### Awareness of Cancer Types and Cancer Statistics

Participants identified a wide variety of cancers, particularly skin, breast, lung, brain, colorectal, pancreatic, liver, leukemia, lymphoma, and osteosarcoma. Less frequently mentioned cancers included eye, uterine, and stomach cancer. Some participants shared knowledge of cancer survival rates, cancer prevalence, and the difference in cancer risk based on sex. As stated by one participant, “If you are a woman of a certain age, you might be more at risk for certain cancers.”

#### Knowledge of Cancer Diagnosis and Treatment

Most participants associated tumors with cancer, while a few participants recognized that cancer could be asymptomatic. A few participants displayed knowledge about the diagnostic process, including the primary site of cancer, staging, and the biopsy process. Participants discussed cancer treatments and their side effects and cited chemotherapy as the most common treatment. Cancer was described as incurable and potentially recurring.

### Negative Perceptions of Cancer

Participants associated cancer with negative emotions. Cancer was described as “scary,” “bad,” and “sad” and associated with fear, suffering, and the loss of hope. Additionally, participants discussed the negative physical and social economic impacts of cancer. Participants described cancer as life-threatening, life-altering, causing physical changes such as hair loss and causing death. Cancer was described as costly, a cause of lost time with family and friends, and by one participant as a “waste of time.”

### Awareness of Cancer Prevention

Participants identified behavioral, physiological, and environmental risk factors for cancer. Individual behavioral factors included sun safety (such as sunscreen use), smoking or tobacco use, diet, alcohol use, drug use, and maintaining healthy habits. Other behavioral factors include engaging in cancer screenings, keeping up to date on vaccines, and medical checkups. Participants stated that physiological factors influence cancer risk, including genetics, age, family history, infection, stress, and viral infection. Environmental factors cited include radiation, asbestos, chemicals, and radon.

### Engagement in Cancer Prevention

#### Interest in Learning About Cancer and Cancer Prevention

Participants expressed interest in learning about cancer and cancer prevention and stated that it was important to learn about cancer. However, some participants expressed no interest. Participants shared that their desire to learn about cancer may be influenced by a personal connection to someone who has experienced cancer, such as family members or friends.

#### Engagement in Cancer Prevention Behavior

Participants reported engaging in multiple types of cancer prevention behaviors, including using sunscreen, maintaining a healthy diet, exercising, and avoiding alcohol, tobacco, and drugs. Sunscreen use and avoiding alcohol were the two most described behaviors, and most participants reported prevention behavior in three areas: eating healthy, exercising, and using sunscreen. Additionally, participants stated that their behavior was influenced by parents, peer support, peer pressure, and family connection to cancer.

#### Attitudes Toward Cancer Prevention

The majority of participants agreed that cancer prevention was important, and some participants agreed that engagement in these behaviors lowers cancer risk. However, participants also acknowledged that engaging in preventative behavior may not prevent cancer.

### Facilitators and Barriers for Action

#### Facilitators—Reasons for Learning About Cancer or Engaging in Preventative Behavior

Participants expressed interest in cancer education and preventative behaviors for personal health reasons and to expand their current understanding of cancer. Personal health factors included a desire to prevent cancer, stay healthy, prevent other diseases or conditions, and act before it is “too late.” In addition, participants wanted to expand their current understanding of cancer to build knowledge, find cure(s), build awareness, and educate others.

#### Barriers—Limiting Factors to Cancer Education or Preventative Behavior

Participants identified several barriers to learning about cancer and engaging in preventive behavior. Some participants perceived cancer as low risk, stating, “people just assume it’s not going to happen to them.” Participants also expressed that preventative behavior has a small impact on overall cancer risk, so they “don’t even bother.” Some participants stated that they forget to engage in preventative behavior, such as wearing sunscreen, while one participant expressed that cancer prevention information could be easily forgotten. Lack of prior experience with cancer was described as a barrier to engaging with cancer content. One participant stated, “some people haven’t had like family members have cancer, so they don’t know as much about it and just don’t think it’s a big deal, whereas some people have had it happen to family members.” A general lack of knowledge about cancer prevention was also cited as a barrier to preventative behavior. Participants discussed the desire to fit into cultural and social norms as a barrier to engaging in preventative behavior. Examples of cultural and social norms included the view that “sunscreen is bad for you” and “if you’re darker-skinned, like you don’t need to wear sunscreen.” Participants described emotional behaviors to engaging in cancer education and prevention, including the futility of prevention behavior, comfort level with talking about cancer, and the frequency they learn about cancer (“getting tired of topic”). One participant stated that “people are just like really uncomfortable talking about medical stuff and stuff like that. So a lot of people would not be interested.” Another participant claimed that “too much, too soon would kind of like turn us away from the topic” and “you get told it so much that you almost start to like not believe it.”

### Preferences for Cancer Education

#### Method of Learning About Cancer

Participants discussed various methods for cancer education, such as presentations given by a cancer survivor or an expert in the field, videos, websites, podcasts, social media, video games, educational games, television, advertisements, and field trips. Learning at school, self-study, or learning via family, friends, and doctors were discussed. Learning through online videos, presentations, and at school were preferred, followed by educational games and social situations. Some participants stated that their learning preference depended on the content delivered.

#### Learning Online and Misinformation

Online videos, websites, and social media were identified as potential sources of misinformation. One participant highlighted how “some people might not know what they’re talking about, maybe, and you might be getting false information.” Participants were concerned about social media being an untrustworthy source for medical or cancer information.

#### Desired Features of Cancer Education

Participants preferred learning about cancer in an interactive, entertaining, relatable, and engaging manner. Participants desired fun, interesting, and knowledge-building content, visuals, and graphics to aid in learning, and a personal connection to the material.

## Discussion

Study participants reported being exposed to cancer content and topics from a wide variety of sources. Many participants stated that cancer is multifactorial in origin and relies on both physiological and behavioral factors. However, few participants recognized that cancer could be asymptomatic, and many identified tumors as the primary symptom of cancer development. This suggests that there is a lack of effective cancer and cancer prevention education for adolescents. Inadequate knowledge of the signs and symptoms of cancer can lead to delayed presentation and diagnosis of cancer, resulting in worse outcomes [15]. Providing adolescents with the learning opportunities needed to influence healthy behaviors that persist into adulthood and the integration of formal instruction may better inform adolescents of the diagnostic process associated with cancer [16].

Adolescents in this study associated cancer with negative emotions, views, and attitudes. Most frequently, participants described cancer as life-altering, costly, and potentially fatal. Emotional states influence adolescents’ self-efficacy and can assist in improving individual learning regarding cancer prevention and education [17]. By addressing the emotions associated with cancer, adolescents may be better equipped to process cancer education and their experiences with cancer, leading to improved overall cancer awareness.

Participants identified numerous risk factors for the development of cancer, including behavioral, physiological, and environmental elements. They recognized individual factors such as sun exposure, smoking/tobacco use, diet, alcohol, and drug use that can contribute to cancer development. However, some participants shared that they lacked cancer prevention knowledge. The World Health Organization (WHO) predicts that about 30 to 50% of cancer deaths could have been prevented by avoiding key risk factors, along with early detection and diagnosis [18]. Therefore, being aware of the signs and symptoms of cancer can reduce cancer mortality if cases are detected and treated in the early stages of cancer development [19]. Educational cancer interventions for adolescents will result in increased knowledge of cancer prevention and could potentially have life-long impacts, improving survival rates [4, 20, 21].

Many participants acknowledged the importance of cancer prevention and expressed interest in learning more about prevention. Some reported engaging in preventative behaviors, such as sunscreen use, healthy diet, and exercise. Many of these healthy habits and behaviors emerge during adolescence as adolescents develop independence and begin to take responsibility for their lifestyle choices around this time [22]. However, risky behaviors may also develop during adolescence and carry on into adulthood. Dietary choices, sun exposure, and exposure to carcinogens are common cancer risk factors in adolescence [23–25]. In 2020, 4.7% of middle school students reported using electronic cigarettes, and 6.7% reported using any tobacco product [26]. Targeting these cancer prevention areas is essential to lowering adolescent cancer risk. Engagement of adolescents through education is ideal due to their ability to actively learn at this stage of life and provides researchers and educators with an optimal opportunity for education and engagement in preventative behaviors [11].

Participants reported wanting to engage in preventative behavior, but shared numerous barriers to proper cancer education and preventative behaviors. Barriers to preventative behaviors included social and cultural norms, lack of knowledge, discomfort when talking about cancer, and forgetfulness. These perceptions are mirrored by national data showing higher cancer death rates for African Americans and increased incidence rates for several cancers among rural populations [27]. Cancer disparities in the United States are well established and should be considered when developing cancer education materials and programs. For instance, factors such as lower socioeconomic status (SES), genetics, decreased treatment adherence, and other health conditions may contribute to higher cancer incidence and mortality in certain populations [28]. Adolescents in this study supported the importance of culturally competent educational materials that address population-specific concerns and barriers.

Adolescents reported using online resources such as websites, videos, and social media to learn about cancer. However, youth also identified these resources could be sources of potential misinformation and emphasized the importance of trustworthy sources when learning about cancer online. Adolescents utilizing the internet as a source of information has been described in previous literature and our study further supports these findings [29]. Implementation of a personalized, interactive educational program using already established resources will allow for the promotion of cancer prevention.

### Limitations

Adolescent perspectives and preferences may have limited generalizability to the overall adolescent population in the United States, as data were collected from one Wisconsin high school and middle school. In addition, the sample was mostly comprised of white middle school students in a specific geographic region. Due to the COVID-19 pandemic, data collection was canceled at multiple sites, including high schools, thus leading to a majority middle school-aged sample. Additionally, due to the seriousness and sensitive nature of discussing cancer, some adolescents may have felt uncomfortable sharing their perspectives or knowledge in a focus group in front of their peers and the study team. Future research should examine the perspectives and preferences of a more representative sample and incorporate other data collection measures.

## Conclusion

This study explored adolescents’ perceptions about cancer and cancer prevention and their preferences for receiving cancer education. Adolescents learned about cancer through a variety of sources, including family and friends, healthcare professionals, online resources, such as social media, websites, and videos, and written materials. Adolescents preferred interactive, relatable, and engaging educational content delivered through online videos, personal presentations, and educational games. Many expressed an interest in learning about cancer and cancer prevention and recognized the importance of cancer prevention. However, some adolescents identified personal barriers to engaging in preventative behaviors and avoiding cancer risks. Adolescents cited forgetfulness, lack of knowledge, desire to fit into social norms, and lack of personal connection to cancer as barriers to engaging in healthy behaviors. Educational interventions and programs should address barriers to preventative behavior and target specific areas of cancer prevention, such as having a healthy diet, limiting sun exposure, and avoiding tobacco products. Educating adolescents on cancer prevention is essential to lowering their risk of cancer in the future.

## Supplementary Information

Below is the link to the electronic supplementary material.

Supplementary file1 (DOCX 21 KB)

## Data Availability

Verbatim quotes from focus group participants have been included in the manuscript. To facilitate openness, transparency, and reproducibility of our research, we have attached the focus group discussion guide used for this study in Appendix A.
