# Supplementary material for: Exploring Youth Perceptions About Cancer Prevention and Preferences for Education: a Qualitative Study
Source: J Cancer Educ. 2021 Aug 13;38(1):50–9. doi: 10.1007/s13187-021-02077-0 (PMC8360774; doi:10.1007/s13187-021-02077-0)
Supplement: Supplementary file 1 — Supplementary file1 (DOCX 21 KB) [file 13187_2021_2077_MOESM1_ESM.docx]

**FOCUS GROUP QUESTIONS**

*BEFORE STARTING, HAND OUT NAME TAGS AND DIRECT STUDENTS TO COME UP WITH FAKE NAMES TO REFER TO EACH OTHER*

**Thank you all for being here. My name is __________, and this is my co-moderator, __________, and we will be leading this focus group today. We are both researchers working with Dr. Abraham at the UW School of Pharmacy.**

**The purpose of this focus group is to learn your thoughts about cancer and cancer prevention. We are very interested in hearing your thoughts and ideas so that we can create better educational materials for people your age. We really want to hear from each of you and encourage everyone here to share their opinions. However, you may choose to pass on answering any questions that you do not feel comfortable with. You may also leave the focus group if you choose to at any time.**

**As mentioned in the consent document, we will be recording this focus group conversation. Your personal information will remain confidential, and none of these records will be linked back to you. It is important that you keep today’s discussion private by not sharing what is talked about by your peers in this room. Please avoid saying your name or the names of others in this group. We also ask that you avoid saying the names of any cities, teachers, schools, classmates, or family members.**

**Lastly, since this conversation will be recorded, we ask that you try to speak loudly and avoid speaking over each other as much as possible. What questions can we answer before we start the focus group?**

*START THE AUDIO RECORDING DEVICE*

**Before we begin asking questions, let’s go clockwise around the table with everyone sharing their fake name for the group.**

*START WITH MODERATOR NAME AND HAVE STUDENTS STATE FAKE NAMES*

**Thank you, now let’s begin with some general questions about cancer.**

GENERAL:

**What does “cancer” mean to you?**

**What do you think of when you hear the word cancer?**

**What types of cancer have you heard about? And what do you know about those cancers?**

*ASK THE CO-MODERATOR IF THERE IS ANYTHING THAT THEY WANT TO ADD*

CANCER RISK/PREVENTION

**What are some risk factors for developing cancer?** *(LIST RISK FACTORS ON BOARD/PAPER)*

**Do you try to avoid these risk factors? Why or why not?**

**How do you avoid these risk factors?**

**How much do you think cancer prevention and avoiding risk factors will help lower your chance of getting cancer when you are older?**

**Can you think of any ways to prevent cancer?**

**How important is cancer prevention to you?**

*ASK THE CO-MODERATOR IF THERE IS ANYTHING THAT THEY WANT TO ADD*

CANCER EDUCATION EXPERIENCE

**Where have you learned about cancer before today? What methods did you use to learn about cancer?** *(PROBE: HAVE YOU LEARNED THROUGH BOOKS? PAMPHLETS? VIDEOS? ETC.)*

**What did you like or dislike about these methods for learning about cancer?**

**Is there one specific method that you liked most? Least?**

*ASK THE CO-MODERATOR IF THERE IS ANYTHING THAT THEY WANT TO ADD*

CANCER EDUCATION PREFERENCES

**Would people your age be interested in education about cancer? Why or why not?**

**Would people your age be interested in education about cancer prevention specifically? Why or why not**

**Do you have any thoughts on how people your age would prefer to learn about cancer and cancer prevention?**

**Would you use websites and online videos to learn about cancer and cancer prevention? Why or why not?**

**Would you use any kind of social media to learn about cancer and cancer prevention? Why or why not? Which form of social media would you prefer and why?** *PROMPT: WOULD YOU USE SNAPCHAT? TWITTER? FACEBOOK? INSTAGRAM? ANYTHING ELSE?*

**How would you feel about learning about cancer and cancer prevention from an expert at a presentation at your school? How about a presentation by someone who has experienced cancer personally?**

**Would you use podcasts to learn about cancer and cancer prevention? Why or why not?**

**Would you use educational video games to learn about cancer and cancer prevention? Why or why not?**

**Of all the ways of learning about cancer that we’ve talked about today, which would you prefer? Are there any ways we haven’t mentioned that you’d prefer?**

*ASK THE CO-MODERATOR IF THERE IS ANYTHING THAT THEY WANT TO ADD*

GAME DEVELOPMENT

**Now let’s do an activity. We can split up into two groups or all work together, depending on your preference.**

**I’d like you all to take about 10 minutes to design an educational video game that would teach other people your age about cancer and cancer prevention. The purpose of the game will be to teach people who play it how to lower their risk of getting cancer and to make healthy choices that will help prevent cancer.**

**Your video game should include the following things:**

1. **Characters: The characters can be anything you want- skateboarding dogs, a talking moon, books that have come to life- anything!**
2. **Setting: Where is this game taking place? Is it at school? At the grocery store? In outer space?**
3. **A storyline: Something should happen to the characters- they have to do things.**
4. **Your video game should teach about cancer and cancer prevention.**

**Don’t worry if you don’t know a lot about cancer; you can still design the game outline as best you can without knowing all the facts about cancer.**

*HAND OUT PAPER AND PENCILS/MARKERS FOR GAME DESIGN (OR HAVE STUDENTS USE WHITEBOARDS IF THAT IS AN OPTION)*

**I’d like you to take notes about your videogame on the paper provided. And again, the goal is to design a game that will teach other people your age about cancer and cancer prevention.**

**Any questions?**

IF SPLITTING INTO GROUPS:

**Okay, let’s divide into two groups. This isn’t a competition between the groups- we just want to give you the opportunity to work together on this activity.**

*MODERATOR SPLIT UP GROUP IF NECESSARY*

**We’ll give you 10-15 minutes to complete this task.**

GIVE STUDENTS 10-15 MINUTES TO WORK ON GAME. ENCOURAGE STUDENTS TO EXPAND ON GAME ELEMENTS AND ASK THEM QUESTIONS ABOUT WHAT THEY’RE CREATING.

**Okay, let’s come back together. Can someone from each group volunteer to describe your videogame? (and draw it on the whiteboard-**IF APPLICABLE)

**Why did you choose that story and those characters for your videogame? Is this something you’d like to play?**

*ASK THE CO-MODERATOR IF THERE IS ANYTHING THAT THEY WANT TO ADD*

*REPEAT WITH BOTH/ALL GROUPS*

**Now I’d like to finish by going around the room one-by-one. Please tells us if there is one new thing you learned today about cancer or cancer prevention. If there is anything else you’d like to share, such as additional suggestions for cancer education or even suggestions about this focus group, feel free to share that as well.**

*COLLECT SHEETS USED TO DESIGN THE GAME.*

**Thank you all for participating in this focus group. All of your responses are very helpful, and the information we gain from these focus groups will help us develop better ways for teens to learn about cancer and cancer prevention.**

*STOP AUDIO RECORDING DEVICE.*

*PASS OUT DEMOGRAPHIC QUESTIONS*
